# Complete mitochondrial genome of the Korean torrent catfish *Liobagrus andersoni* (Siluriformes, amblycipitidae)

**DOI:** 10.1080/23802359.2016.1242389

**Published:** 2016-11-12

**Authors:** Seungki Lee, Ji Hyung Kim, Ha Yeun Song

**Affiliations:** a Biological and Genetic Resources Assessment Division, National Institute of Biological Resources, Incheon, Republic of Korea;; b Infectious Diseases Research Center, Korea Research Institute of Bioscience and Biotechnology, Daejeon, Republic of Korea;; c Genetic Resources Team, National Marine Biodiversity Institute of Korea, Seocheon-gun, Republic of Korea

**Keywords:** Siluriformes, amblycipitidae, *Liobagrus andersoni*, Korean torrent catfish

## Abstract

The Korean torrent catfish (*Liobagrus andersoni*), an endemic species in Korea, is a member of the family Amblycipitidae. Herein, we report the first sequencing and assembly of the complete mitochondrial genome of *L. andersoni*. The complete mitochondrial genome is 16,514 bp long, consisting of 13 protein-coding genes, 22 tRNA genes, two rRNA genes, and a control region. It has the typical vertebrate mitochondrial gene arrangement. Phylogenetic analysis using mitochondrial genomes of 21 species showed that *L. andersoni* was clustered with *L. marginatoides* and *L. nigricauda*. This mitochondrial genome provides potentially important resources for addressing taxonomic issues and studying molecular evolution.

The Korean torrent catfish, *Liobagrus andersoni* (Regan 1908), is a small benthic species in the family Amblycipitidae that is endemic to Korea. This species is allopatrically distributed in the upper and middle parts of the Imjin and Han rivers (Kim & Park 2007; Kim et al. 2015). To date, there has been very little molecular and genetic research on this species. To the best of our knowledge, this is the first study to determine the complete mitochondrial genome of *L. andersoni*, and to analyze the phylogenetic relationship of this species among Siluriformes fishes.

The *L. andersoni* specimen used in this study (standard length, 88.1 mm) was collected from the Imjin River, Jeongseon-gun, South Korea (37.12N, 128.38E). The specimen was deposited in the National Institute of Biological Resources (NIBR, Voucher No. NIBRGR0000166819). Genomic DNA from muscular tissues was sequenced and assembled using the Illumina Hiseq 4000 sequencing platform (Illumina, San Diego, CA) and *SOAPdenovo* assembler at Macrogen Inc. (Korea), respectively. The obtained complete mitochondrial genome was annotated using MacClade ver. 4.08 (Maddison & Maddison 2005) and DNASIS ver. 3.2 (Hitachi Software Engineering). Experiments were conducted in accordance with the Guidelines of Animal Ethics published by the NIBR.

The complete mitochondrial genome of *L. andersoni* (GenBank accession no. KX767082) is 16,514 bp long, and includes 13 protein-coding genes, 22 tRNA genes, and two rRNA genes. The *ND6* gene and five tRNA genes are encoded on the light strand. The overall base composition of the heavy strand is 30.37% A, 28.76% C, 15.81% G, and 25.04% T. Similar to the mitogenomes of other vertebrates, the AT content is higher than the GC content (Saccone et al. 1999). All tRNA genes can fold into a typical cloverleaf structure, with lengths ranging from 67 to 75 bp. The 12S rRNA (953 bp) and 16S rRNA genes (1,667 bp) are located between tRNA^Phe^ and tRNA^Val^ and between rRNA^Val^ and tRNA^Leu(UUR)^, respectively. Of the 13 protein-coding genes, 12 start with ATG; the exception being *COI*, which starts with GTG. Seven of the 13 protein-coding genes were shown to terminate with incomplete stop codons, T (*ND2, COII, COIII, ND3, ND4,* and *Cytb*) and TA (*ATP6*), whereas the remaining six genes ended with complete stop codons of TAA or TAG. A control region (897 bp) is located between tRNA^Pro^ and tRNA^Phe^.

Phylogenetic trees were constructed by the maximum-likelihood method with 1000 replicates using MEGA 7.0 software (MEGA, Philadelphia, PA) (Kumar et al. 2016) for the newly sequenced genome and 20 further complete mitochondrial genome sequences downloaded from the National Centre for Biotechnology Information. We confirmed that *L. andersoni* is clustered with *L. marginatoides* (Jia et al. 2013a) and *L. nigricauda* (Jia et al. 2013b) and rooted with the other Amblycipitidae species (Figure 1). This mitochondrial genome provides potentially important resources for addressing taxonomic issues and studying molecular evolution.

**Figure 1.**
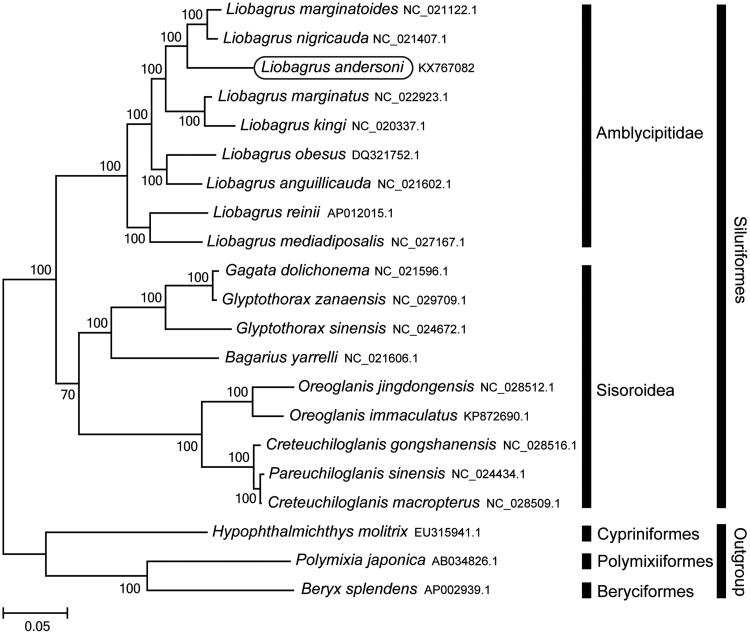
Phylogenetic position of *Liobagrus andersoni* based on a comparison with the complete mitochondrial genome sequences of 20 species. The analysis was performed using the MEGA 7.0 software. The accession numbers for each species are indicated after the scientific names.
